# Modulation of early maize seedling performance via priming under sub-optimal temperatures

**DOI:** 10.1371/journal.pone.0206861

**Published:** 2018-11-05

**Authors:** Gokhan Hacisalihoglu, Sarfo Kantanka, Nathan Miller, Jeffery L. Gustin, A. Mark Settles

**Affiliations:** 1 Florida Agricultural and Mechanical University, Department of Biological Sciences, Tallahassee, FL, United States of America; 2 University of Wisconsin, Department of Botany, Madison, WI, United States of America; 3 University of Florida, Horticultural Science Department, Gainesville, FL, United States of America; Brigham Young University, UNITED STATES

## Abstract

Seeds planted in early spring frequently experience low temperature stress in the soil during germination and early plant growth. Seed pretreatments such as priming have been shown to ameliorate the negative effects of cold soil in some crops. However, the potential beneficial effects of priming have not been widely investigated for *Zea mays* (maize). To investigate seed priming effects, 24 diverse maize inbred lines were primed using a synthetic solid matrix, Micro-Cel E, and then exposed to 10°C soil conditions. Six DSLR cameras captured time lapsed images of emerging seedlings. Manual scoring was used to determine treatment effects on three seedling emergence metrics. Chilling substantially reduced total emergence for two of 24 genotypes evaluated. For these genotypes, priming provided protection allowing nearly full emergence. Priming significantly reduced mean emergence time and increased the emergence uniformity of chilling sensitive genotypes. The results suggest that the cold sensitive genotypes may benefit from priming pretreatment. Kernel density, weight, oil, protein, and starch traits, as determined by single-kernel near infrared spectroscopy, were not correlated with seedling emergence traits supporting a conclusion that early seedling performance cannot be determined from these maize kernel characteristics.

## Introduction

Chilling stress is one of the major limitations on kernel emergence especially for C4 plants such as maize [1]. Maize is of tropical origin and is considered to have limited tolerance to low temperature stress. However, substantial maize cultivation occurs at temperate latitudes where the crop is exposed to a colder environment early in the season. Planting dates for the US Corn Belt range from April to early May when cold soil temperatures can induce chilling stress. Maize kernel emergence and growth is impaired at suboptimal temperatures with mild chilling being 10–15°C and severe chilling being below 6°C [2–4]. Kernel weight and composition can influence tolerance to cold stress, particularly in sweet corn, with heavier kernels, containing higher starch, showing better germination [5–7]. In the field, small kernels may have lower emergence under cold conditions [8]. In some studies, there is evidence that kernel traits are not associated with differences in germination under cold conditions [9].

**Fig 1:**
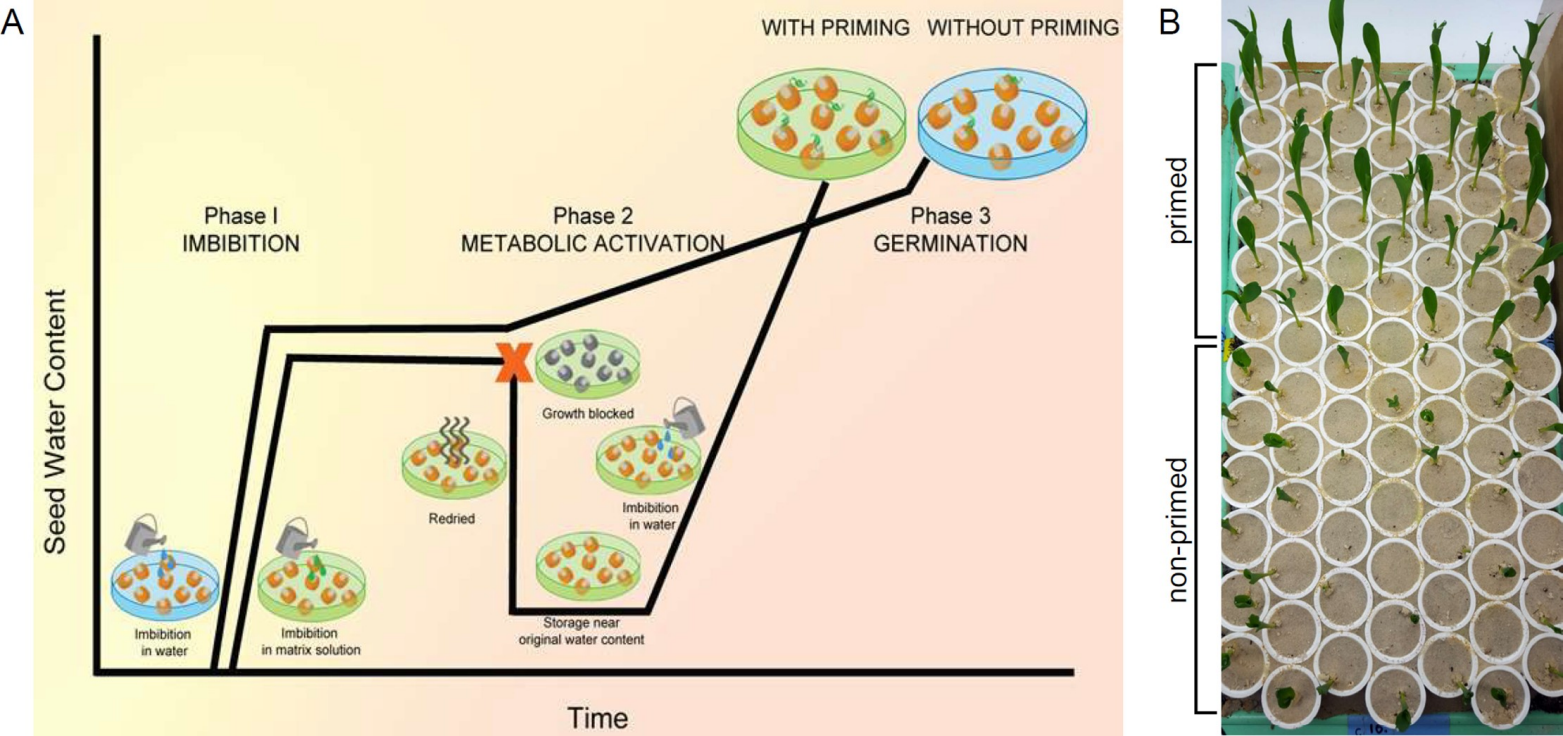
Overview of seed priming and time-lapse imaging emergence assay. (**A**) Model of germination stages in non-primed and primed kernels. (**B**) Seedling growth of MO37W primed kernels (top half of tray) and non-primed kernels (bottom half of tray) six days after transfer to 25°C.

Seed priming can increase emergence rate, early seedling growth and stand establishment in many plant species [10]. Priming treatments incubate seeds in a hydrating solution to allow imbibition and initiation of the first stages of germination (Fig 1A). Just prior to radical protrusion, seeds are removed from the solution and dried back to the approximate initial mature dry weight [11].

There are a number of priming methods including hydropriming, solid matrix priming and osmotic priming that have been used to improve emergence and seedling growth. [12, 13]. Hydropriming involves soaking seeds in water for a short period of time, often overnight, where water uptake is not controlled by the priming solution. Osmotic priming incubates seeds in solutions that contain osmotically active compounds such as salts, sugars, or polyethylene glycol (PEG). The concentration of the osmolytes in the priming solution determine the water potential between the solution and the seed and regulate water uptake. Similarly, solid matrix priming uses chemically innert solid carriers (e.g. Micro-Cel E) with negative water potential to regulate water uptake. Both solid matrix and osmotic priming slow the rate of imbibition as compared to hydropriming and allow for additional time to repair cellular membranes and cellular damge (10) and have been show to improve emergence of cold stressed seeds. Yadav et al. [14] demonstrated that chemical seed priming increased sub-optimal temperature tolerance of Jatropha by promoting faster and more uniform seedling emergence. Chen et al. [15] found that spinach seeds primed with PEG 8000 had improved emergence in 5°C soils.

Studies on the effect of priming on low temperature tolerance of maize are frequently limited to an individual hybrid genotype within a study to enable comparisons among priming methods. Multiple studies have compared hydropriming with other priming agents with hydropriming consistently showing less improvement in germination or emergence [16–22]. Micronutrient or osmotic priming agents can improve germination and early seedling development at sub-optimal temperatures [19, 20, 22, 23]. Some hormones, reducing/oxidizing chemicals, chitosan, and even *Moringa oliferia* leaf extracts have been shown to increase germination or seedling emergence in low temperatures [16–18, 20, 21, 24, 25]. Two *shrunken2* sweet corn varieties also showed improved emergence under cold stress after solid matrix priming using the seed disinfectant, sodium hypochlorite [26]. These studies suggest that many kinds of priming agents could improve soil emergence characteristics for typical cold temperatures in northern latitudes at planting. However, the performance of priming has not been compared directly between diverse maize genotypes.

The specific objectives of this study were to: 1) evaluate the effects of solid matrix priming on the emergence characteristics of a diverse set of maize inbred lines under 10°C soil treatment; and 2) evaluate the interaction between priming and kernel composition on seedling emergence. The effects of priming were determined by comparing multiple seedling performance characteristics including emergence percentage, emergence timing, and emergence uniformity derived by scoring time lapsed images of seedling emergence assays.

## Materials and methods

### Plant materials

Kernels of 24 maize (*Zea mays L*.) inbred lines selected from the parental lines of the Nested Association Mapping (NAM) population were acquired from the United States National Plant Germplasm System, North Central Region Plant Introduction Station [27]. Each inbred line was bulked with controlled pollinations in the April-July 2015 field season at the University of Florida Plant Science Research and Education Center in Citra, FL using standard agronomic practices. Kernels were harvested, dried to approximately 10% moisture content, and stored at 10°C and 50% relative humidity until use.

### Kernel priming treatment

Kernel priming used Micro-Cel E (Imersy Filtration, San Jose, CA), which is non-soluble, synthetic calcium silicate. Solid matrix priming was completed as described previously with some modifications [12, 28, 29]. Samples were primed by mixing 1 g of kernels, 0.5 g Micro-Cel E, and 1.5 mL water, which was held in containers with lids for 36 h at 25°C. The 36 h priming duration gave the fastest germination time among 12 h to 48 h treatments for preliminary tests with B73 and B97 primed kernels. Primed kernels were dried back to their approximate original weight by placing the kernels in a single layer in front of a large fan overnight (Fig 1A).

### Emergence assay

Forty-two kernels of each genotype were used for chilling and optimal treatment conditions. Kernels were planted in heavy-duty emergence trays (Carolina Biological Supply Company, Burlington, NC) (10 x 20 in) filled with professional growing mix soil (Sungro Hort, Agawam, MA), watered to saturation, and leveled across the surface of the tray. For the chilling temperature treatment, soil trays were watered to saturation and held at 10°C prior to planting.

To track individual kernels, honeycomb structures were constructed by gluing together 84 schedule 40 polyvinyl chloride (PVC) 5.1 cm couplings (Lasco fittings, Inc., Brownsville, TN) in a 7 x 12 array using medium strength PVC glue. An impression of the honeycomb form was made in the soil, and one kernel per cell was placed on the soil surface with the abgerminal side facing away from the soil. The form was placed over the indexed kernels and sieved premium play sand (Quikrete, Columbus, OH) was added to fill the couplings (Fig 1B). Trays were placed in 5 cm of 10°C or ambient temperature water for 30 min to fully hydrate the sand. For the 10°C treatment, trays were immediately transferred to a Percival unit set at 10°C for 5 d.

Emergence trays were then placed on shelving in a walk-in growth chamber at 25°C with continuous fluorescent illumination positioned 70 cm above the shelf. Images were automatically acquired by Canon EOS Rebel T5 DSLR cameras. The cameras were attached to the shelving and positioned 45° from horizontal using custom braces. From this fixed position, one camera could image two trays. Cameras were tethered to a laptop running 64bit Ubuntu operating system 16.04 LTS and raw images were acquired every 30 min using gPhoto2, stored locally, and uploaded to the CyVerse (http://www.cyverse.org/) data infrastructure for storage. Image names encoded date, time, and camera information. The date and time of planting, initial watering, and transfer to 25°C were recorded along with the number of images to determine the time to emergence for each seedling. Seedling emergence was determined for each kernel by manual inspection of the time lapsed images and correcting for the start time of image capture. The emergence times for the individual kernels imaged are in S1 Table.

### Physiological parameters and kernel composition

Maize kernel composition traits were estimated by single-kernel NIR using custom calibrations [30, 31]. Each kernel was analyzed by the skNIRS four times generating four kernel weights and four spectra. Each kernel spectrum was fit to Partial Least Squares (PLS) linear regression models that transformed the spectral data into predictions for relative kernel starch, protein, and oil content as well as kernel density and volume. Data for the four skNIR replications were averaged for each kernel. For comparison with seedling emergence, kernel data were averaged by genotype according the treatment factors listed in the tables.

### Data analysis

Emergence percentage, time, and uniformity were used to evaluate the effects of priming and low temperature. Emergence percentage measured the number of seedlings that emerged relative to the number planted. Emergence time measured the mean number of hours seedlings took to emerge after being transferred to the 25°C growth condition. For cold-treated seedlings planted in 10°C, emergence time was measured starting 5 d after planting, when trays were transferred to 25°C. Emergence uniformity was determined by fitting a logistic curve to the cumulative percent emerged per hour using the R package ‘nls’ [32, 33]. The logistic function was defined as:

$$y=\left(\frac{\theta 1}{1+e^{-(\theta 2+\theta 3x)}}\right)+\varepsilon$$

where *y* is the cumulative percent emerged, *x* is emergence time, θ1, θ2, *and* θ3 are the model parameters estimated by maximum likelihood, and ε is residual error. Emergence uniformity is represented by the rate of increase or θ3 in the fitted model. Standard error of the fitted curves were measured as the deviance of the fitted values divided by the degrees of freedom of the residuals.

To isolate priming treatment effects from seed quality, the relative ratio of 10°C to 25°C plantings was calculated for relative emergence percentage (rEm%), relative emergence time (rEmT), and relative emergence uniformity (rEmU). Analysis of variance (ANOVA) was used to determine the treatment effects [33].

## Results and discussion

### Response of emergence percentage to priming

**Fig 2:**
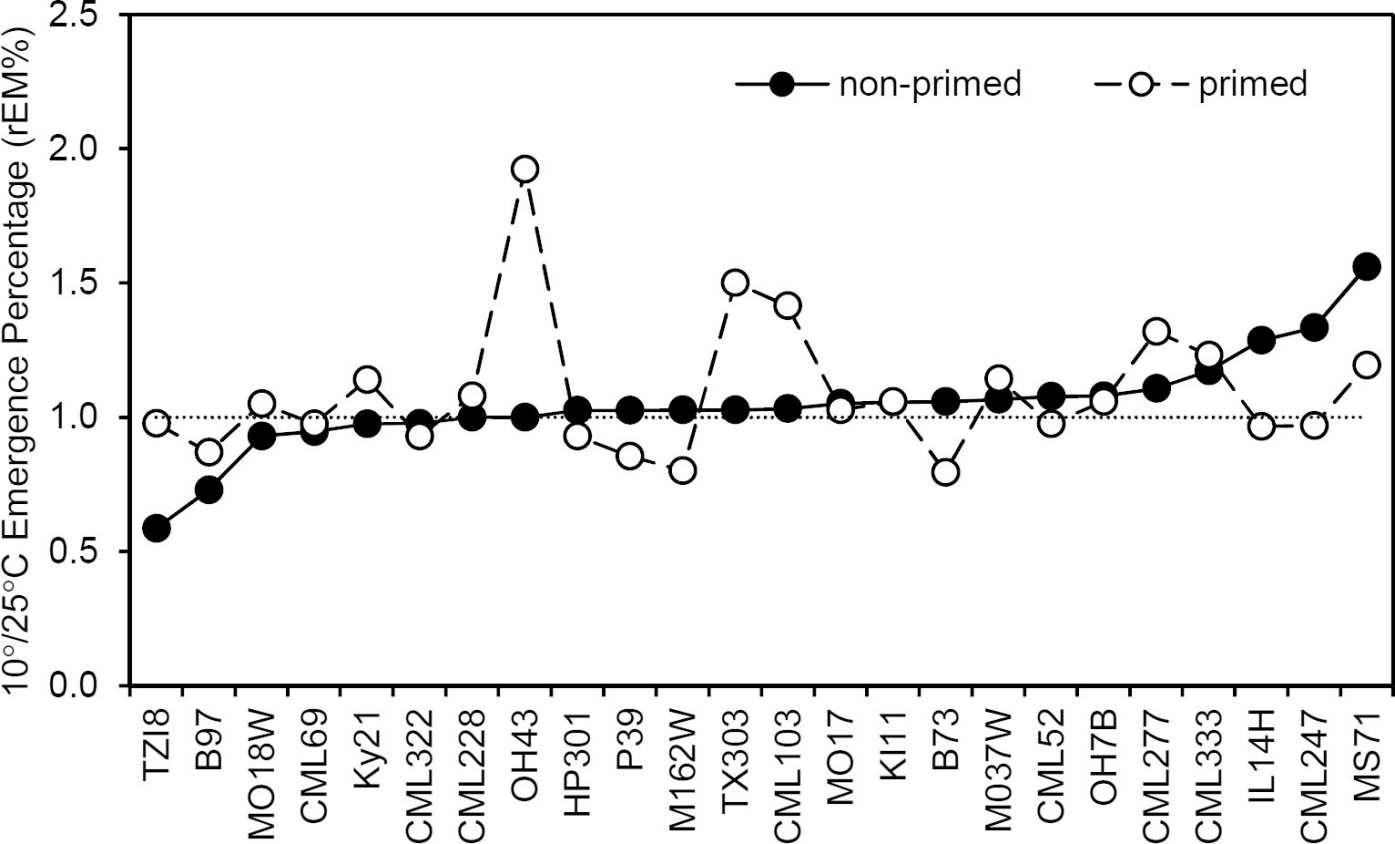
Seed priming may improve emergence percentage for chilling sensitive genotypes. Ratio of 10°/25°C emergence percentage (rEm%) for each genotype under primed and non-primed pretreatments. Inbred lines are ranked based on non-primed rEm%.

Sensitivity to the 10°C soil incubation was evaluated by calculating the relative emergence percentage (rEm%) expressed as a ratio of seedlings emerged at 10°C to seedlings emerged at 25°C. Fig 2 shows rEm% for each genotype under both primed and non-primed pretreatments and ranked according to non-primed rEm%. Across all genotypes neither priming nor chilling had a significant effect on the emergence percentage based on a two-way ANOVA using temperature and priming as the main effects (Tables 1 and 2). However, exposing the kernels to 10°C for 5 d specifically reduced the emergence percentage of two genotypes, TZI8 and B97. For both genotypes, priming pretreatment increased their rEm%. TZI8 emerged at only 58% of control after 10°C soil treatment, while primed kernels emerged at 97%. Cold imbibition of B97 reduce emergence to 73% of control, while primed seed emerged at 89% of control. These data suggest that priming may improve cold tolerance for the genotypes whose total emergence is reduced by 10°C treatment.

**Table 1:** Statistical summary of primed and soil temperature treatments on seedling emergence (Em) traits.

		25°C		10°C	
Em trait	Statistic	Non-primed (NP)	Primed (P)	Non-primed (NP)	Primed (P)
**%**	**Mean**	77	74	81	80
	**SD**^**a**^	20	22	17	15
	**Range**	33–100	10–100	38–100	45–100
**Time (h)**	**Mean**	114	109	97	91
	**SD**	10.6	10.2	10.5	11.9
	**Range**	94.9–134	88.6–131	80.2–119	66.9–112
**Uniformity (θ3)**	**Mean**	0.25	0.22	0.26	0.23
	**SD**	0.09	0.06	0.10	0.10
	**Range**	0.09–0.42	0.09–0.36	0.09–0.43	0.07–0.52

^a^SD, standard deviation

**Table 2:** Two-way ANOVA for emergence (Em) traits.

Em trait	Factor	df^a^	Mean Sq^b^	F	pr(>F)^c^
**%**	**Priming**	1	0.005	0.23	0.63
	**Temperature**	1	0.02	1.09	0.29
	**Interaction**	1	0.002	0.09	0.76
**Time (h)**	**Priming**	1	925	7.9	0.006
	**Temperature**	1	9148	78.3	<0.001
	**Interaction**	1	12	0.1	0.75
**Uniformity (θ3)**	**Priming**	1	0.013	1.74	0.19
	**Temperature**	1	0.005	0.69	0.41
	**Interaction**	1	0.001	0.24	0.62

^a^df, degrees of freedom

^b^Mean Sq, mean squares

^c^pr(>F), p-value of factor effect

### Response of emergence time to priming

Kernels planted in 10°C for 5 d emerged 17 h sooner than 25°C controls (Table 1). A multi-factor ANOVA using temperature, priming, and their interaction as modeled effects showed both priming and temperature were significant (Table 2). Temperature had the largest effect on mean emergence time, most likely due to the 5 d of 10°C imbibition prior to transfer into 25°C. Priming significantly reduced emergence time by 6.5 h (Tables 1 and 2). However, the effect of priming on emergence time was equivalent in both 10°C and 25°C treatments (Table 1). Consequently, the interaction coefficient between priming and temperature was not significant in the ANOVA (Table 2). This analysis shows that priming hastens emergence but does not specifically improve emergence time after 10°C imbibition.

**Fig 3:**
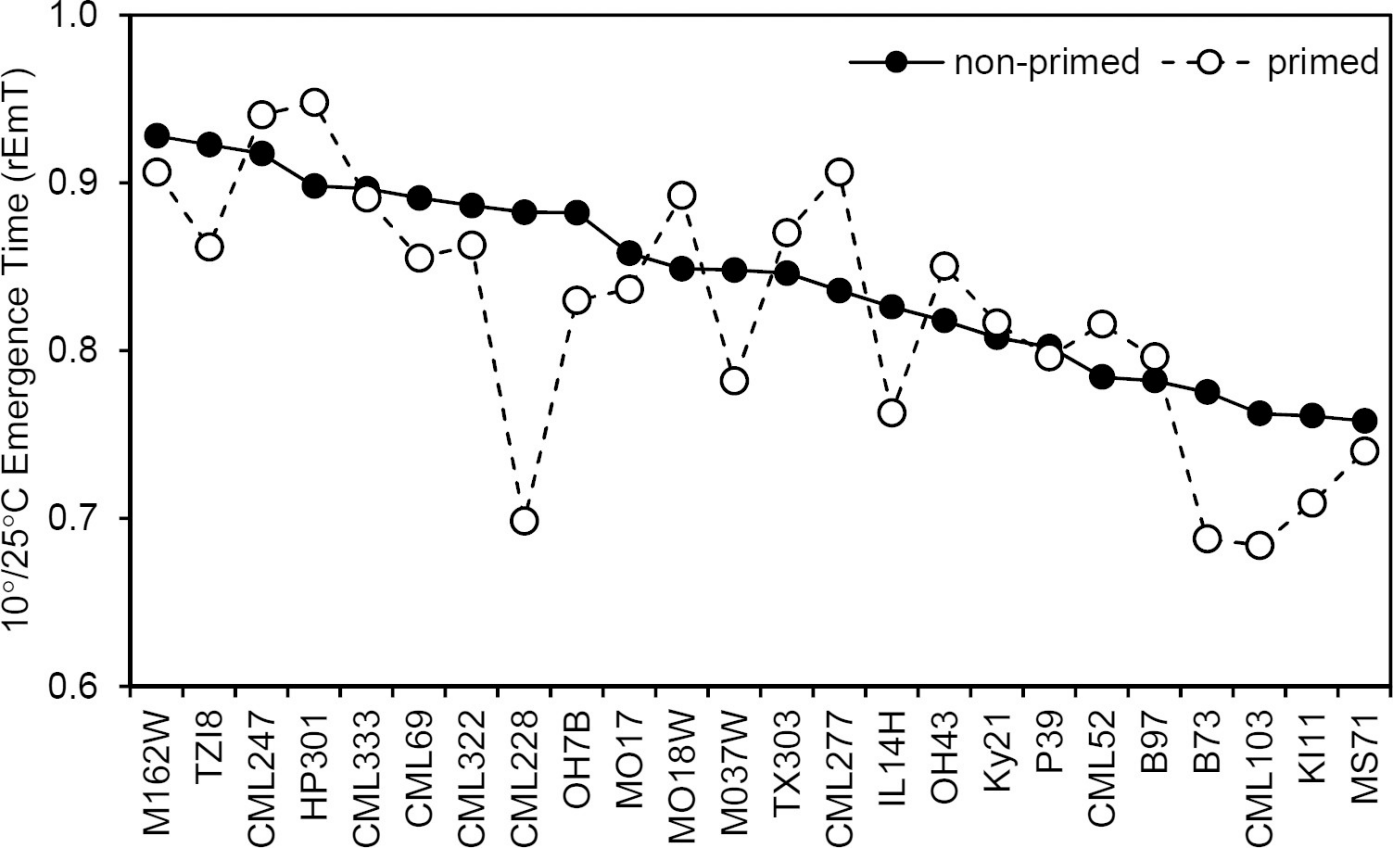
Priming hastens emergence regardless of chilling stress. Ratio of 10°/25°C emergence time (rEmT) for each genotype under both primed and non-primed pretreatments. Genotypes most affected by 10°C imbition have the highest rEmT values. Inbred lines are ranked according to non-primed rEmT.

The relative emergence time ratio (rEmT) of 10°/25°C illustrates the variable effects of priming among the genotypes tested (Fig 3). Since the 10°C-treated kernels emerged sooner after transfer to 25°C than controls, the ratio for all genotypes was less than one. Genotypes with the smallest rEmT emerge most rapidly after the 10°C treatment and can be interpreted to be least affected by the cold treatment. The three genotypes with the highest non-primed rEmT were TZI8, M162W, and CML247 with values of 0.93, 0.92, and 0.92, respectively. TZI8 also had the lowest relative emergence percentage (Fig 2). Priming reduced relative time to emergence and increased relative emergence percentage for TZI8 suggesting some mitigation of the chilling stress in this genotype.

### Response of emergence uniformity to priming

**Fig 4:**
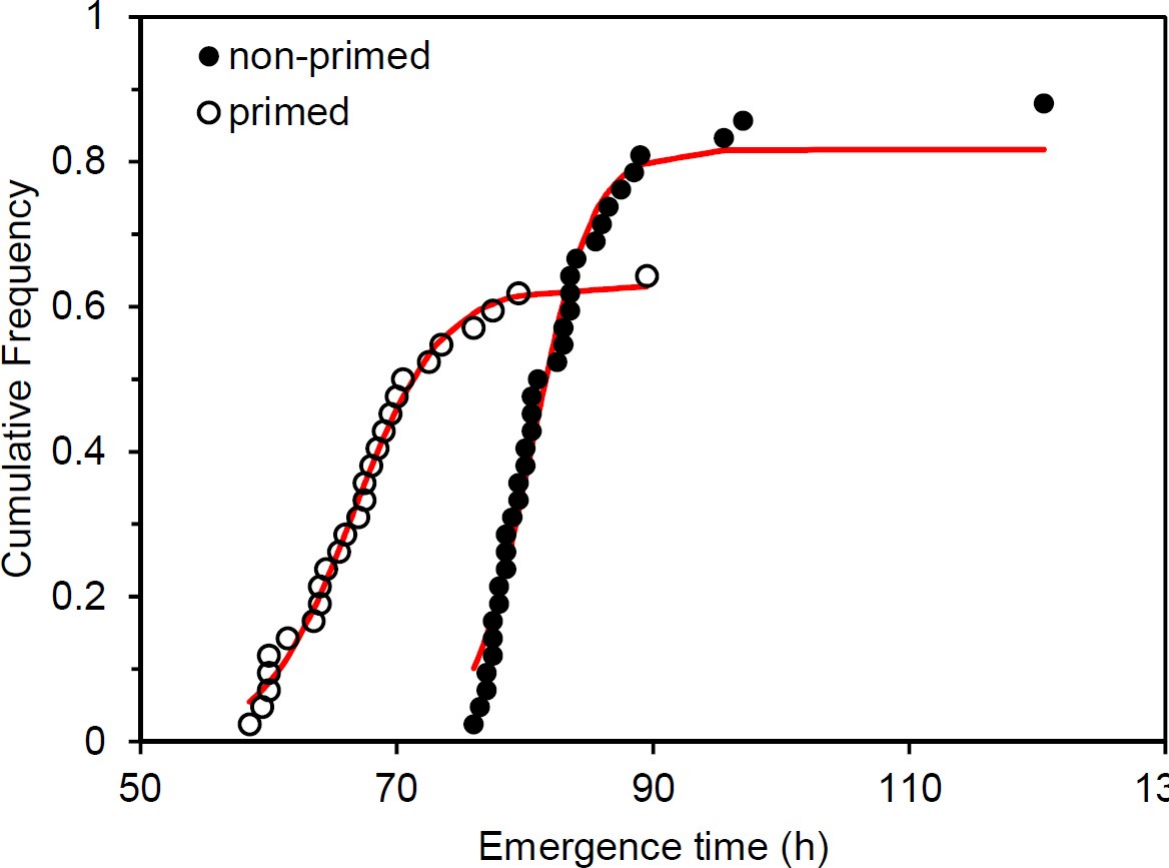
Cumulative seedling emergence can be modeled with a logistic curve. Scatterplot showing observed cumulative frequencies of seedling emergence for primed and non-primed B73 after the 10°C treatment. Logistic fits of each data set are plotted with red lines.

Variation in emergence time is a significant factor in yield with uniform emergence being an important trait for quality field establishment [34]. We modeled emergence uniformity using a logistic growth model fit to the cumulative frequency distributions of emergence for each genotype. Fig 4 displays the observed emergence and fitted logistic models for B73 in primed and non-primed conditions. The fitted values were a good approximation of the observed data for all genotypes with an average standard error of 0.03% emerged per hour and a maximum standard error of 0.06% emerged per hour. The θ3 parameter from the model describes the steepness of the logistic growth curve and was taken as the emergence uniformity trait, with a larger θ3 parameter value indicating a more uniform emergence.

**Fig 5:**
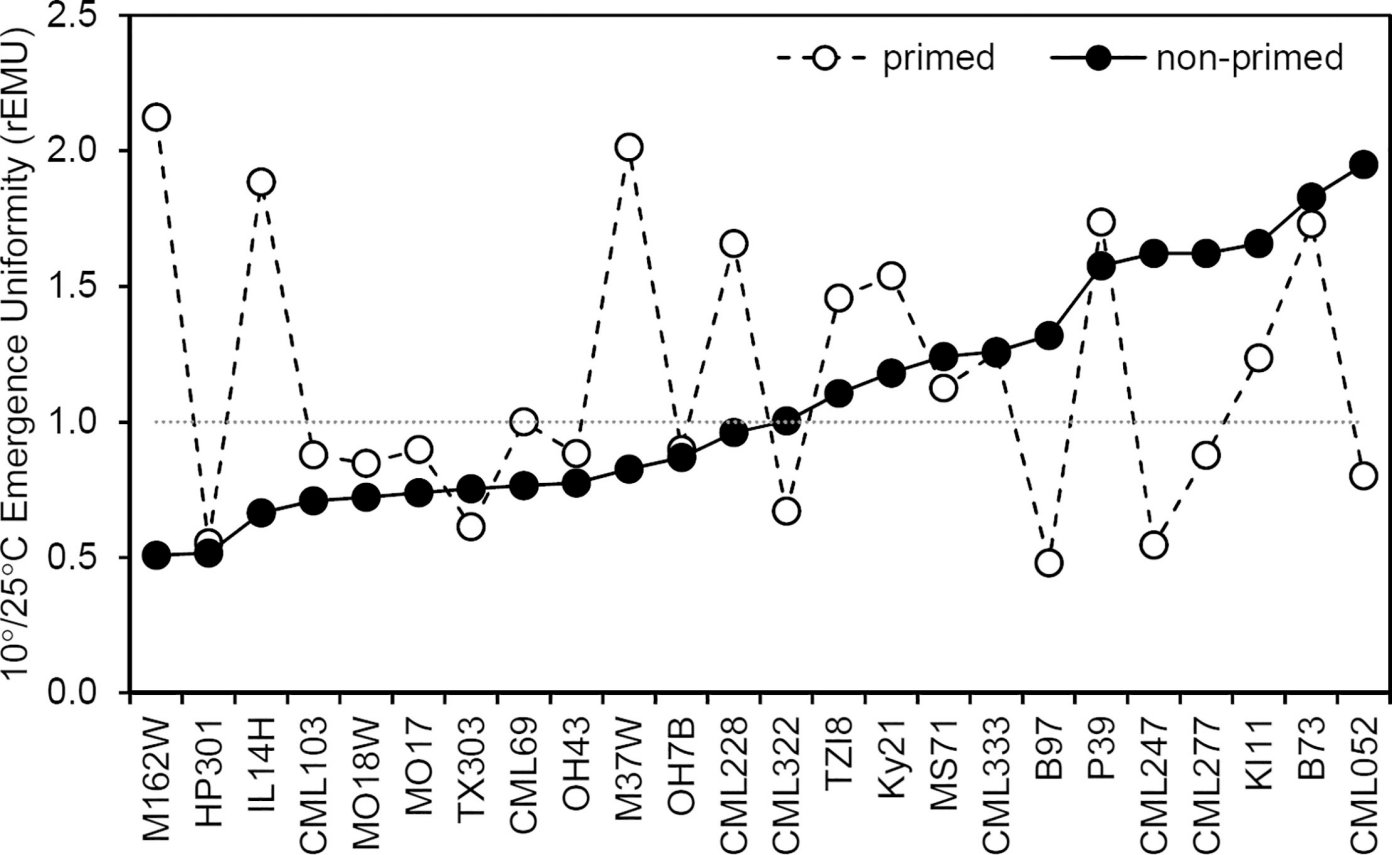
Priming may improve emergence uniformity in chilling sensitive genotypes. Ratio of 10°/25°C emergence uniformity (rEmU) for each genotype under both primed and non-primed pretreatments. Larger rEmU values (>1) indicate more uniform emergence after the 10°C pretreatment. Inbred genotypes are ranked according to non-primed rEmU.

Multi-factor ANOVA using temperature, priming and their interaction as treatments found no significant effects of these treatments on emergence uniformity (Table 2). These statistics show that priming did not impact the uniformity of emergence on the 24 genotypes as a whole. However, emergence uniformity showed a wide range among the genotypes (Table 1). The effects of cold treatment on emergence uniformity were evaluated by calculating the 10°/25°C ratio (rEmU). Fig 5 displays the non-primed and primed rEmU for each genotype and ordered by non-primed rEmU. Twelve of 24 non-primed genotypes had a rEmU below one indicating emergence became more spread after the 10°C cold treatment as compared to control conditions. Interestingly, priming improved relative uniformity in all but one of these cold sensitive genotypes. By contrast, priming reduced rEmU in seven of twelve genotypes whose non-primed rEmU was above one suggesting priming was detrimental to genotypes that had more uniform emergence in cold conditions. While priming does not impact uniformity in general, these data suggest that priming can improve emergence uniformity for genotypes that are sensitive to the 10°C treatment.

### Relationships between emergence traits

Correlation analysis of emergence percentage, time, and uniformity showed that the traits were a largely independent metrics (Table 3). These three traits were weakly correlated with higher emergence percentage associated with faster and more uniform emergence. Other studies found that mean emergence time in cold conditions was related to emergence percentage. Matthews and Hosseini [35] found strong correlations between mean germination time in a 13°C cold test and field emergence percentage. However, more limited genetic variation was tested with only 11 seed lots of commercial hybrids included in the study [35].

**Table 3:** Correlation coefficients (r) among seedling emergence (Em) traits.

Em Trait	%	Time
**Time**	-0.37^a^	
**Uniformity**	0.28^a^	-0.38^a^

^a^False Discovery Rate <0.01 using Benjamini Hochberg adjusted p-values.

Hu et al. [36] monitored the germination of 241 inbred lines from a maize association panel in 8°C and 25°C conditions. In this study, a persistent cold treatment resulted in genotypes with low germination rates also germinating slower (*r* = -0.86). Our 5 d cold treatment (10°C) was followed by transferring kernels to 25°C conditions for emergence and was a more mild stress as compared to Hu et al. [36]. For example, the CML103 and HP301 inbred lines failed to germinate with continuous 8°C, while this study found that the rEM% for CML103 and HP301was not affected by the 5 d, 10°C cold treatment (Table 4). The difference in cold treatments likely accounted for the lower correlation with our chilling treatment between emergence percentage and emergence time.

**Table 4:** Mean values of kernel composition and relative emergence traits.

Genotype	Weight (mg)	Volume (cm^3^)	Starch (%)	Protein (%)	Oil (%)	Density (g/cm^3^)	rEm% NP^a^	rEm% P^b^	rEmT NP	rEmT P	rEmU NP	rEmU P
**B73**	244	217	63.1	11.4	2.43	1.23	1.06	0.79	0.78	0.69	1.83	1.73
**B97**	244	235	58.8	10.5	3.46	1.25	0.73	0.87	0.78	0.80	1.32	0.48
**CML52**	222	184	58.4	11.8	3.55	1.47	1.08	0.97	0.78	0.82	1.95	0.80
**CML69**	228	206	59.2	11.5	3.86	1.50	0.95	0.97	0.89	0.86	0.76	1.00
**CML103**	248	227	59.5	13.0	2.38	1.35	1.03	1.41	0.76	0.68	0.71	0.88
**CML228**	194	184	58.6	11.8	3.78	1.39	1.00	1.08	0.88	0.70	0.96	1.66
**CML247**	156	180	56.5	12.2	3.15	1.34	1.33	0.97	0.92	0.94	1.62	0.54
**CML277**	204	205	58.4	10.9	3.17	1.44	1.11	1.32	0.84	0.91	1.62	0.88
**CML322**	173	180	56.7	12.0	4.54	1.41	0.98	0.93	0.89	0.86	1.00	0.67
**CML333**	242	216	54.1	12.0	5.37	1.53	1.17	1.23	0.90	0.89	1.26	1.25
**HP301**	93.0	78.0	54.1	13.7	3.10	1.40	1.03	0.93	0.90	0.95	0.52	0.55
**IL14H**	178	163	49.8	13.0	5.32	1.50	1.29	0.97	0.83	0.76	0.66	1.88
**KI11**	220	211	59.4	10.6	3.49	1.39	1.06	1.06	0.76	0.71	1.66	1.24
**Ky21**	222	231	59.1	10.1	2.14	1.32	0.98	1.14	0.81	0.82	1.18	1.54
**M37W**	187	206	60.9	10.5	2.43	1.36	1.07	1.14	0.85	0.78	0.83	2.01
**M162W**	242	236	62.5	9.30	3.16	1.37	1.03	0.80	0.93	0.91	0.51	2.12
**MO17**	197	200	54.8	12.6	2.64	1.36	1.05	1.03	0.86	0.84	0.74	0.90
**MO18W**	209	221	60.9	10.2	3.85	1.46	0.93	1.05	0.85	0.89	0.72	0.85
**MS71**	232	222	57.6	12.1	3.29	1.34	1.56	1.19	0.76	0.74	1.24	1.13
**OH7B**	205	230	58.0	12.1	4.34	1.41	1.08	1.06	0.88	0.83	0.87	0.90
**OH43**	209	215	61.5	11.4	2.00	1.35	1.00	1.92	0.82	0.85	0.77	0.88
**P39**	188	166	48.8	13.6	8.47	1.48	1.03	0.85	0.80	0.80	1.58	1.74
**TX303**	257	239	66.2	9.00	2.23	1.40	1.03	1.50	0.85	0.87	0.75	0.61
**TZI8**	215	205	61.6	9.30	3.85	1.51	0.59	0.98	0.92	0.86	1.10	1.46
**Mean**	207	202	58.1	11.6	3.56	1.40	1.05	1.09	0.84	0.82	1.09	1.15
**SD**^**c**^	36	34	3.9	1.3	1.4	0.1	0.18	0.25	0.05	0.08	0.43	0.50

^a^NP, non-primed

^b^P, primed

^c^SD, standard deviation

### Correlation with kernel composition traits and seedling parameters

Few studies have investigated the relationships between kernel composition and seedling emergence. We used a single-kernel NIR platform to determine kernel weight, volume, density as well as the relative levels of protein, oil, and starch prior to priming and planting to investigate the relationships between kernel traits and emergence characteristics (Table 4).

Tables 5 and 6 display the correlation coefficients between kernel composition and seedling emergence. Priming and temperature treatments showed no effect on emergence percentage and uniformity (Table 2); consequently, the measurements of these traits in the four separate conditions were averaged for comparison with the kernel traits (Table 5). While none of the coefficients were significant at a false discovery rate of 0.10, there was a moderate negative relationship between relative kernel protein content and emergence uniformity (r = 0.-46, adjusted p = 0.30) suggesting that higher protein content may reduce uniformity.

**Table 5:** Correlation coefficients (r) among kernel composition and seedling emergence (Em) percentage and uniformity.

Em Trait	Weight	Volume	Starch	Protein	Oil	Density
**%**	0.02	-0.02	-0.05	-0.06	0.25	0.17
**Uniformity**	0.24	0.35	0.35	-0.46	0.09	0.1

No coefficients were significant at a False Discovery Rate <0.10 using Benjamini Hochberg adjusted p-values.

**Table 6:** Correlation coefficients (r) among kernel composition and seedling emergence (Em) time.

Temp.	Kernel Pretreatment	Weight	Volume	Starch	Protein	Oil	Density
**10°C**	**non-primed**	-0.05	-0.06	-0.14	0.01	0.04	0.36
	**Primed**	-0.34	-0.31	-0.28	0.08	0.09	0.37
**25°C**	**non-primed**	0.23	0.11	-0.15	0.13	-0.03	0.11
	**Primed**	-0.1	-0.19	-0.38	0.29	0.12	0.2
**10°/25°C**	**non-primed**	-0.38	-0.24	-0.01	-0.13	0.1	0.39
	**Primed**	-0.37	-0.24	-0.04	-0.16	0.02	0.34

No coefficients were significant at a False Discovery Rate <0.10 using Benjamini Hochberg adjusted p-values.

Seedling emergence time was significantly altered by both priming and imbibition temperature treatments (Table 2). Consequently, correlations between emergence time and kernel composition traits were assessed within each condition (Table 6). Again, kernel composition was not significantly associated with emergence time in any of the tested conditions. The strongest relationship was between starch and emergence time in the 25°C, primed treatment combination (r = -0.38). The negative relationship suggests high starch levels promote earlier emergence in primed kernels. This result is consistent with Wang et al. [37] who reported that time to 50% germination was negatively correlated with starch content in watermelon. Kernel weight showed a weak relationship with relative emergence time under both primed and non-primed treatments suggesting that lighter kernels were somewhat delayed in emergence after cold imbibition.

Among the individual genotypes, priming effects on HP301 were notable. Priming reduced total emergence by approximately 30% in both warm and cold treatments (warm primed/non-primed = 70%; cold primed/non-primed = 63%). In addition, HP301 was only one of two genotypes whose emergence time was delayed by priming (warm primed/non-primed = 0.95; cold primed/non-primed = 0.90). These observations suggest that HP301 was damaged by the priming treatment and needed time to recover before germinating.

In conclusion, this study showed that kernel priming with Micro-Cel E significantly reduced emergence time across most genotypes while emergence percentage and uniformity were not generally affected. Emergence percentage and uniformity appeared to be improved by priming in the subset of genotypes that were cold sensitive. However, we also observed that priming can be detrimental to emergence uniformity in some genotypes. Priming, therefore, may be beneficial only for genotypes with known sensitivity to low-temperature, spring planting conditions typical of the U.S. Corn Belt. We did not detect significant relationships between kernel composition or size with seedling emergence traits.

## Supporting information

S1 Table Underlying data for seedling emergence statistics. (CSV)
